# DAILY PSYCHOLOGICAL EXPERIENCES AND SLEEP HEALTH AMONG AFRICAN AMERICAN FAMILY CAREGIVERS

**DOI:** 10.1093/geroni/igae098.0593

**Published:** 2024-12-31

**Authors:** Elliane Irani, Stephanie Griggs, Zhengyi Chen, Ronald Hickman

**Affiliations:** Case Western Reserve University, Cleveland, Ohio, United States; Case Western Reserve University, Cleveland, Ohio, United States; Case Western Reserve University School of Medicine, Cleveland, Ohio, United States; Case Western Reserve University, Cleveland, Ohio, United States

## Abstract

Extensive research has been conducted on the reciprocal associations between daily psychological experiences and sleep health (i.e., sleep satisfaction, daytime alertness, sleep timing, efficiency, and duration) in the general population. However, little research has focused on whether these associations vary for African American family caregivers who experience unique stressors and report lower sleep satisfaction and shorter sleep duration than other groups. We investigated how daily psychological experiences, including positive and negative affect, general stress, caregiving stress, and positive experiences, impact same-night sleep in African American family caregivers, and how nightly sleep affects next-day experiences. African American family caregivers (n=31) wore a research-grade actigraphy device day/night and completed daily diaries at bedtime and waketime over 7 days. Multilevel models evaluated within-person associations between daily psychological experiences and sleep health adjusted for participants’ age, prior-day outcome, and weekend (vs. weekday). Participants in the study were mostly women (80.6%), with an average age of 58.3 years. Most participants were caring for a parent (77.4%). On days when participants reported higher-than-average prior night sleep satisfaction, they reported higher-than-average positive experiences and affect, and lower-than-average negative affect. Daytime alertness was linked to higher positive experiences and affect, and lower negative affect and general stress. Results for the reversed direction revealed that negative affect, general stress, and positive experiences predicted next-day daytime alertness. Findings support the psychological health benefits of sleep in the context of African American family caregivers’ daily life and suggest that interventions should focus on improving sleep health to enhance daily psychological experiences.

